# Editorial

**Published:** 1861

**Authors:** 


### Editorial.

An Explanation.—The omission of the April number of the Examiner requires an explanation. The manuscript for the original department of that number was placed in the hands of the printer about the usual time, and the first "form" of eight pages was worked off in a few days. We had no intimation but that the work was going on as usual, and being fully occupied with other professional engagements, we did not stop to enquire until near the last of the month; when, much to our surprise, we found the war excitement had stopped all work in that printing establishment, and caused its doors to be closed—at least, for the present. Before we had time to effect an arrangement with any other publisher, we were called suddenly to Springfield to act on a Board of Medical Examiners, and detained there one week. Reaching home on the 2d of May, a satisfactory arrangement was immediately perfected with the present publishers, and the work pushed forward as rapidly as possible. But, inasmuch as the time for the April number had been entirely lost, we have thought it proper to date the present number for April and May, both; promising our readers that the deficiency of the April number shall be fully made up by improvements in the subsequent numbers of the present year.

Prospects for the Future.—One of the principal objects that induced us to commence the publication of the Chicago Medical Examiner, was to establish a Medical journal here, which should not only constitute a medium through which the medical writers of the Northwest could communicate their thoughts and observations, but which should, also, faithfully reflect the doings of the Medical societies, colleges, hospitals and dispensaries of this city, as fully as the medical journals of New York and Philadelphia do, the doings of the various medical institutions of their respective cities. An early failure in the health of Dr. E. A. Steele, who was associated with us as Assistant Editor, has hitherto prevented us from carrying out our original design as perfectly as we had desired. From the present date, however, his place will be supplied by Frank W. Reilly, M. D., a recent graduate,—energetic and industrious, a ready writer, an accurate reporter, and well versed in all the duties devolving on an assistant in editorial labors. With his aid, we hope to make such improvements in all departments of the Examiner as will be gratifying to its readers.

Surgeons and Assistant Surgeons in the Army.—For the information of that large class in the profession who are volunteering their services as surgeons, we would state, that the laws of this State provide for only *one* Surgeon and *one* Assistant Surgeon to each *regiment* of volunteers.

The six regiments first called into service from this State, in compliance with the requisition of the general government, are now organized and fully supplied with medical officers. A recent act of the Legislature provides for the immediate organization of ten additional regiments, to be completely organized, and drilled for thirty days, and held in readiness for any future demand that may be made, either by the State or national governments. Surgeons and Assistant Surgeons to these ten regiments are to be appointed solely by the Colonel of each regiment, and the law does *not* provide for an examination before such appointments, as was the case with the first six regiments. This is much to be regretted; for personal observation has fully satisfied us that only a small part of those who are proffering their professional services, have any adequate idea of the duties of a medical officer of the army. Many think of nothing but the ability to cut off limbs and dress mangled wounds, forgetting, entirely, the fact that, in all armies, disease destroys ten for every one that falls on the field of battle. Whoever becomes attached to a regiment of volunteers as a medical officer, should be thoroughly awake to all those sanitary measures that are calculated to ward off disease, or mitigate its violence. The necessity of subjecting all candidates to a rigid examination, will be abundantly apparent by the results developed in the following report:

REPORT OF THE

### BOARD OF MEDICAL EXAMINERS

TO THE LEGISLATURE.

The Board of Physicians and Surgeons appointed by the Legislature of the State of Illinois, to examine such candidates as might present themselves for the office of Surgeon or Assistant Surgeon in the six regiments of volunteers called into active service by the authority of this State, would respectfully report as follows, viz:

The members of the Board assembled at the State House, in Springfield, Ill., April 26th, 1861, at 9½ o'clock A. M.

Present—Drs. G. W. Stipp, of Bloomington; W. M. Chambers, of Charleston; N. S. Davis, of Chicago; and Charles Ryan, of Springfield. Absent—Dr. Carpenter, of St. Clair County.

A quorum being present, the Board was organized by the appointment of Dr. G. W. Stipp, Chairman, and Dr. N. S. Davis, Secretary. The following rules were then adopted for the government of the Board in the transaction of its business:

1st.—The Board shall hold three sessions daily, commencing at the hours of 8½ A. M., 2 P. M., and 8 P. M.

2d.—Each candidate for examination shall furnish satisfactory evidence that he has received the degree of *Doctor of Medicine* from a regular Medical College in good standing in the profession.

3d.—Each candidate shall furnish satisfactory evidence that he possesses a good moral character.

4th.—The examination of each candidate shall have special reference to the four following topics, viz: Surgical Anatomy, Practical and Operative Surgery, Practical Medicine, and Hygiene.

5th.—Immediately after the examination of each candidate the result of such examination shall be determined by ballot, without debate—and *two* negative votes shall be sufficient to reject the candidate.

The whole number of candidates whose names were presented to the Board and placed on file for examination was *fifty-two;* of whom fifteen failed to make their appearance before us.

Of the *thirty-seven* who were duly examined, *nine* were found qualified for the office of *Surgeon*, and were furnished with certificates accordingly, to-wit:

David Prince, M. D., of Jacksonville; Horace Wardner, M. D., of Chicago; Henry W. Davis, M. D., of Paris; S. T. Trowbridge, M. D., of Decatur; A. W. Heise, M. D., of Joliet; James H. Faris, M. D., of Danville; Christopher Goodbrake, M. D., of Clinton; Sanford Bell, M. D., of Springfield; and Dr. Evarts, of Quincy.

And *twenty-one* were furnished with certificates, as qualified for the office of *Assistant Surgeons*—to-wit:

D. W. Young, M. D., of Aurora; Samuel C. Plummer, M. D., of Rock Island; A. E. Goodwin, M. D., of Rockford; John L. Teed, M. D., of Mendota; B. F. Stephenson, M. D., of Petersburg; John M. Phipps, M. D., of Charleston; Wm. F. Cady, M. D., of Rock Island; Charles Davis, M. D., of Alton; James Hamilton, M. D., of Springfield; J. W. Van Valza, M. D., of Freeport; P. H. Bailhache, M. D., of Springfield; Conrad Dumreicher, M. D., of Chicago; Thos. Wilkins, M. D., of Vandalia; Daniel Stahl, M. D., of Quincy; J. R. Gore, M. D., of Chicago; Geo. H. Knapp, M. D., of Jerseyville; H. A. Buck, M. D., of Marengo; Samuel M. Hamilton, M. D., of Monmouth; E. A. Steele, M. D., of Chicago; Edgar Winchester, M. D., of Elgin; George W. Crossley, M. D., of Princeton.

Being fully aware that our citizen soldiery, summoned, as they have been, suddenly from their various avocations, to the hardships and exposures of the camp and the field, would be in far more danger from attacks of ordinary diseases, than from injuries on the field of battle, we have deemed it our duty, as Examiners, to inquire as carefully concerning the views and acquirements of each candidate in relation to the treatment of disease and such hygienic measures as aid in preserving health, as in relation to their surgical knowledge and skill. And though the very short time intervening between the passage of the law creating the board, and its meeting for business, may have prevented some members of the profession in the more distant parts of the State from appearing before it, yet we feel much pleasure in assuring you and the people of the whole State, that among those we have deemed it our duty to recommend as *Surgeons*, our patriotic volunteers will find as skillful Physicians and Surgeons, and as faithful and devoted friends as they could possibly desire. And though we have been less exacting in the standard of acquirements fixed for the office of *Assistant Surgeons*, yet we have intended to give certificates to such *only*, as would do ample credit to themselves, and full justice to those who might need their assistance. Deeming it necessary that a Surgeon or Assistant Surgeon in the army, should be thoroughly acquainted, not only with the nature and treatment of those diseases to which all armies on active duty are liable, but also with all those cases connected with personal habits, topographical conditions, and climatic changes, which tend to engender disease, we have been reluctantly constrained to withhold certificates of recommendation from some, who are justly regarded in civil life as good operative Surgeons and honorable Physicians.

In conclusion, we cannot too warmly commend the considerate care manifested by the Legislature, in the effort to secure for those of our fellow citizens, who have temporarily abandoned the arts of peace for the defense of our government and the integrity of our country, the most reliable Medical and Surgical aid that the State can afford.

We trust that the example now set, will be followed on all similar occasions, if the calamities of war should call for additional regiments, and consequently additional medical officers. All of which is respectfully submitted.

SPRINGFIELD, *May* 1*st*, 1861.

N. S. DAVIS,
GEO. W. STIPP,
WM. M. CHAMBERS,
CHARLES RYAN,
} *Board of Examiners.*

APPOINTMENT.—Ralph N. Isham, M. D., Professor of Surgical Anatomy and Operations of Surgery in the Medical Department of Lind University, has received the appointment of Surgeon to the Marine Hospital in this city. Dr. Isham is a gentleman whose professional attainments, pleasing address, and brilliant style of operating, have won for him no mean reputation, both in the amphitheater and in private practice; and we congratulate him, not less than Uncle Sam, on the increased sphere of usefulness and experience this places at his disposal.

——Students of the Medical Department of Lind University have now the advantage of the wards of two of the most extensive Hospitals in the Northwest, in addition to a large Dispensary practice, at which cliniques are given twice a week during the entire year. Cliniques at the Mercy Hospital, under the services of Professors Davis and Andrews, are given regularly four times per week, during the summer course.

OBITUARY.—The profession in the northwest has been called on to lament the loss of one of its most valuable members, in the death of DR. E. J. FOUNTAIN, of Davenport, Iowa, in the thirty-third year of his age. He was engaged at the time of his death, which took place on the 29th of March, in the prosecution of experiments on the chlorate of potassa—a subject which had engaged much of his attention, and on which he had written several very interesting papers. He was a frequent contributor to the current medical literature, a successful practitioner, an enthusiastic and self-sacrificing student, and occupied a place in the profession which will not be easily filled.

AMERICAN MEDICAL ASSOCIATION.—The annual meeting of this Association appointed to be held in the city of Chicago, on the first Tuesday in June next, is hereby postponed until the first Tuesday in June, 1862, on account of the extremely disturbed condition of the whole country.

CHICAGO, *May* 1*st*, 1861.

N. S. DAVIS,<br>

J. W. FREER,<br>

E. ANDREWS,<br>

DELASKIE MILLER,<br>

J. BLOODGOOD,<br>

THOS. BEVAN,<br>

H. W. JONES,<br>

*Committee of Arrangements.*

Personally, it has been with extreme reluctance that we have assented to the above notice postponing the regular annual meeting of the American Medical Association. But such postponement having been unanimously advised by leading members of the profession, in all sections of the Union, we have felt constrained to yield to the sad necessities of the times. Having all our lives combatted *sectional* prejudices, and advocated a catholicity of sentiment which would embrace our whole country, not only in medicine, but also in religion, politics, and social life, we had fondly hoped to meet our professional brethren from the North, South, East and West, in the same fraternal spirit that has characterized the meetings in years gone by. But we must wait—and while we wait, pray, that before the first Tuesday in June, 1862, every part of our great country may be reposing peacefully under the folds of the old "Star Spangled Banner," and contentedly enjoying the blessing of the mildest and most liberal government ever established among men.

BELLEVUE HOSPITAL COLLEGE.—The importance of clinical instruction, and the value of these bedside teachings, are being rapidly recognized and appreciated by the profession; and we hail the recent act of the New York Legislature, in chartering a new Medical School in connection with Bellevue Hospital, as another step in the right direction. Students will discriminate in favor of institutions, the precepts of whose lecture-rooms are emphasized by practice at the bed-side.

Homœopathy in the Michigan University.—The following letter fully explains itself, and our readers will be gratified by its perusal.—[*Ed. of Examiner.*]

University of Michigan,

*April 19th*, 1861.

Prof. N. S. Davis, M. D.,

*Dear Sir :*—In your journal for March, which has just come to hand, the statement is made that you "learn from the Detroit *Free Press* that the Legislature of Michigan, during its recent session, passed a law, requiring the establishment of a chair of Homœopathy in the Medical Department of the State University, at Ann Arbor."

I write to inform you that this is a mistake, entirely, and to request you, which you will, no doubt, be happy to do, to correct it. I am sorry to learn, as I do now for the first time, that the *Free Press* was led into such an error. It must have corrected it very soon, as it commented approvingly upon the action of the Legislature in *not* passing such an act.

The facts in this case are simply these:

The Homœopathic practitioners in the State, since their defeat a few years ago, in attempting to have their system taught in the University, have been putting forth every possible effort, as we have since learned, in a secret way, to secure the election of persons to the Legislature, favorable to that system, and its introduction into the University.

Among others they succeeded in getting into the Legislature and upon the Committee on Education, a man who now calls himself a Homœopathic physician, but who, not long since, was a professor in the Eclectic Medical College at Cincinnati. This man, aided by a very active *lobby* of Homœopathic physicians and others, by means of the grossest and most shameless misrepresentations, whispered quietly into the ear, and at last embodied in a report, made considerable headway with members.

A bill providing for the introduction of two Homœopathic professors into the University was introduced into the lower house, which, after discussion and the exposure by documentary evidence of many of the false statements made in the report by which the bill was attempted to be sustained, was *lost* for want of a sufficient number of votes.

The friends of the measure, abandoning the idea of mingling Homœopathic teaching with that of legitimate medicine in the same school, introduced another bill providing for the establishment of a *separate school* at any town in the State *other*

*than Ann Arbor.* This passed the lower house, but after discussion and a report thereon in the Senate, was lost in that body by a decisive vote. So all Homœopathic measures were defeated, instead of a law establishing a chair of Homœopathy in the Medical Department of the University being passed.

Your article very properly states that the Constitution of the State places the University entirely under the control of the Board of Regents, and that a few years ago, after thoroughly investigating the claims of Homœopathy to be represented in the University, they refused to recognize it.

By giving place to the above, you will do justice to the Legislature of Michigan, and to legitimate medicine, besides obliging much,

Your humble servant,

A. B. PALMER.

DEWITT COUNTY MEDICAL SOCIETY.—The society met in annual session at the office of Dr. Goodbrake in Clinton, on Tuesday, the 2d day of April. Dr. John H. Tyler in the chair.

The minutes of the previous meeting were read and approved.

On motion of Dr. Edmiston, Dr. E. W. Gideon was invited to participate in the proceedings of this meeting.

The following gentlemen were then elected officers for the ensuing year:

President—Dr. Z. H. Madden.
Vice-President—Dr. Thos. W. Davis.
Treasurer—Dr. T. K. Edmiston.
Secretary—Dr. C. Goodbrake.
Dr. J. H. Tyler,
Dr. John Wright, } Censors.
J. C. Ross,

Delegates to the Illinois State Medical Society—Dr. John Wright, Dr. B. S. Lewis, and Dr. Christopher Goodbrake. Alternates—Drs. Hunt, Adams, and Davis.

Delegates to the American Medical Association—Dr. John H. Tyler, and Dr. Z. H. Madden. Alternates—Drs. Edmiston and Richards.

The President elect then took the chair, and made a few very appropriate remarks.

Dr. J. H. Tyler, the retiring President, then delivered his valedictory address; when on motion of Dr. Goodbrake, the thanks of the society were tendered to the Doctor, and a copy of his address requested for publication.

Dr. Tyler reported a case of Endo-Carditis.

Dr. Madden reported a case of Uterine Hemorrhage.

Drs. Edmiston, Adams and Davis, were appointed essayists for the next meeting.

Diptheria was chosen as the subject for discussion at the next meeting of the society.

On motion of Dr. Wright, it was ordered that the proceedings of the meeting be published in the *Central Transcript;* also in the *Chicago Medical Journal*, and the MEDICAL EXAMINER.

On motion the society adjourned to meet in quarterly session, at Santa Anna, on the first Tuesday in July next.

C. GOODBRAKE, M. D., Secretary.

PERSONAL.—Prof. C. D. Meigs, for twenty years the most popular teacher in the United States, at the close of the recent session of the Jefferson Medical College, resigned his seat in the Faculty of that institution, to the great regret of his colleagues, the profession generally, and a host of alumni and pupils.

Prof. Meigs has been in active practice for upwards of fifty years, and a teacher for nearly half that period. As practitioner, lecturer and author, he has enjoyed a wider and more brilliant reputation, his classes have been larger, and his success greater than, perhaps, that of any other American physician—and, in a very great degree, this has been deservedly so.

He retires, we understand, to his country seat, Hamanassett, near Philadelphia, there to engage in literary and agricultural pursuits, and the composition of a long contemplated history of medicine. The kind wishes and warm sympathies of innumerable friends follow him in his retirement.

A suitable expression of sentiment was entered upon their record by the Faculty on receiving his resignation; and on commencement-day, the graduating class presented him with a portrait of himself, by Waugh.

——Dr. Wm. V. Keating, of Philadelphia, has been chosen to fill the chair of Obstetric Medicine thus vacated. Dr. K. is very highly spoken of by the *North American*, as an obstetric practitioner of enlarged experience, and of great readiness as a lucid and pleasant teacher. He was formerly connected, for a number of years, with the Philadelphia Medical Association, as lecturer on midwifery and diseases of women and children.

——The honorable post of Physician-Extraordinary to Queen Victoria, recently made vacant by the lamentable death of Dr. Baly, has been assigned to Dr. William Jenner, Physician to University College Hospital, and author of several works on fever, etc.

——Dr. Thomas Harris, at the time of his death the oldest Surgeon in the American Navy, died in Philadelphia, March 4th, of apoplexy. Dr. H. was a graduate of the University of Pennsylvania, in the days when there were giants—Rush, Dorsey, Wistar, Barton, Physick,—at its head, and had, for several years, been at the head of the Medical Bureau at Washington.

A Board of Medical Examiners will convene at the Naval Hospital in New York, on the 1st prox., for the examination of applicants for admission into the Naval Medical Staff. Those gentlemen who so successfully lobbied against the appointment of an Examining Board, under the Ten Regiment Bill, might find it to their interest to protest against this also.

The Manuscript of Prof. Andrew's Lecture on *Military Surgery* was handed in too late for the printers to assign it its proper position, under the head of "Original Contributions." We are happy, however, to be able to announce that this is only the first of a series on this engrossing topic, which will be given in our pages during the summer course of the Lind School.

Of 101 applicants for commissions as Surgeons and Assistant Surgeons examined by the New York Board, thirty-nine passed as Surgeons and thirty-seven as Surgeons' Mates.

A Suggestion.—Of the hundred and fifty or two hundred physicians in our own State, and about quadruple that number in the entire North-West, who have volunteered their professional services to the army, not over two out of five will probably be availably used in the camp or field. But we may suggest to the disappointed ones, that fully as much good may be done, and as much patriotism evinced, though in their own home spheres and plain citizens' garb, by quiet but active exertion in directing and stimulating the efforts of citizens to supply the troops with additional hospital conveniences and comforts. The proper Department will see to the furnishing of necessary medicines, etc.; but in the unavoidable confusion and hurry, attendant upon so great and sudden a revolution from a peace to a war establishment, many important details will be overlooked, and much will necessarily devolve upon private individuals. And the preparation of lint and bandages will *not* include all that will be found useful in this department.

Purulent, or Egyptian ophthalmia, is a disease among the most troublesome and common in armies, and the southern portion of this State is one of its favorite habitats. It is asserted that 30,000 cases of this disease occurred in the Prussian army in eight years, and that the Belgian army suffered still more from it. Wounds of the eye, and various other forms of ophthalmia, form also a large per cent. of the cases. *Green silk eye-shades*, as recommended by Col. Anderson, with elastic bands, will be found especially useful, and may be got up in considerable numbers.

*Splints*, of all sizes and shapes, may be prepared. *Field Stretchers*, formed like a hand-barrow, six and a half feet long, and thirty inches wide, with strong canvas bottoms, and projecting handles—can be made by any carpenter. *Saddler's Silk*, for ligatures and sutures; *pins* of various sizes; soft, clean *sponges* (there cannot be too many of these. The inoculation of the virus of erysipelas by indiscriminately dressing all patients with the same sponge, cannot be too sedulously guarded against.) *Towels*, both coarse and fine, as well for ordinary bathing purposes as for hospital use; *Cotton batting* for compresses, fracture-dressings, etc.; *thin, light flannel shirts*, long and loose, for the sick, with drawing-strings at the neck, instead of buttons; and shorter and more closely-fitting ones for the active soldiery; large-sized *list* or *cloth slippers; lime* and *lemon juice* in strong black bottles, tightly corked with practicable corks, *i. e.*, those not requiring a cork-screw to extract; *vaccine virus*, fresh and healthy, either in vials, dissolved in glycerine, or in the usual form, in wax,—for the vaccination of the unprotected at discretion ; *adhesive* and *isinglass plaster* may also be prepared, or purchased, and sent. *Cap-covers* of light, white cotton cloth, with capes falling down over the shoulders, will be very useful under the Southern sun. The *Havelock cover* is the best we have seen, and patterns of it may be obtained by writing to the Rev. Dr. Ryder, of St. Paul's Church, in this city, who will be happy to send them by mail to applicants. *Light Ambulances*, with two or three stretchers, mounted on springs and drawn by two horses, are quite serviceable on the field of battle, or in conveying the wounded and sick from outposts to the hospitals or heavy ambulances.

To the above, the physician of every village sending a company, might add such articles as he deemed serviceable, and set the ladies to work at once before their energies are completely exhausted scraping lint and rolling bandages—of which the venerable Dr. Mott says there have been enough prepared in New York city alone, to last a seven years' war. Such supplies, properly packed in strong tool-chests, and with a list of contents tacked to the inside of the lid, should be sent to the Surgeon of the regiment to which the company is attached. No fear but their contents will be acceptable.

